# Systematic Review: Patient Perceptions of Monitoring Tools in Inflammatory Bowel Disease

**DOI:** 10.1093/jcag/gwaa001

**Published:** 2020-01-24

**Authors:** Thomas M Goodsall, Richard Noy, Tran M Nguyen, Samuel P Costello, Vipul Jairath, Robert V Bryant

**Affiliations:** 1 Gastroenterology Department, John Hunter Hospital, Newcastle, Australia; 2 School of Medicine and Public Health, The University of Newcastle, Newcastle, Australia; 3 Radiology Department, John Hunter Hospital, Newcastle, Australia; 4 Robarts Clinical Trials Inc., London, Ontario, Canada; 5 Department of Gastroenterology, The Queen Elizabeth Hospital, Adelaide, Australia; 6 Faculty of Health Sciences, School of Medicine, University of Adelaide, Adelaide, Australia; 7 Department of Epidemiology and Biostatistics, Western University, London, Ontario, Canada; 8 Department of Medicine, Division of Gastroenterology, Western University, London, Ontario, Canada

**Keywords:** Acceptability, Inflammatory bowel disease, Monitoring tools, Patient preference, Tolerability

## Abstract

**Background and Aims:**

Inflammatory bowel disease (IBD) is a lifelong disease requiring frequent assessment to guide treatment and prevent flares or progression. Multiple tools are available for clinicians to monitor disease activity; however, there are a paucity of data to inform which monitoring tools are most acceptable to patients. The review aims to describe the available evidence for patient preference, satisfaction, tolerance and/or acceptability of the available monitoring tools in adults with IBD.

**Methods:**

Embase, Medline, Cochrane Central and Clinical Trials.gov were searched from January 1980 to April 2019 for all study types reporting on the perspectives of adults with confirmed IBD on monitoring tools, where two or more tools were compared. Outcome measures with summary and descriptive data were presented.

**Results:**

In 10 studies evaluating 1846 participants, monitoring tools included venipuncture, stool collection, gastrointestinal ultrasound, computed tomography, magnetic resonance imaging, wireless capsule endoscopy, barium follow-through and endoscopy. Outcome domains were patient satisfaction, acceptability of monitoring tool and patient preference. Noninvasive investigations were preferable to endoscopy in nine studies. When assessed, gastrointestinal ultrasound was consistently associated with greater acceptability and satisfaction compared with endoscopy or other imaging modalities.

**Conclusions:**

Adults with IBD preferred noninvasive investigations, in particular gastrointestinal ultrasound, as compared to endoscopy for monitoring disease activity. When assessing disease activity, patient perceptions should be considered in the selection of monitoring tools. Further research should address whether adpoting monitoring approaches considered more acceptable to patients results in greater satisfaction, adherence and ultimately more beneficial clinical outcomes.

## Introduction

Inflammatory bowel disease (IBD) is a lifelong disorder with increasing global prevalence (1). Patients are burdened by high rates of surgery, reduced quality of life, and social and occupational dysfunction (2,3). Improving outcomes for patients with IBD necessitates treatment directed toward amelioration of inflammation (4–6). Attainment of mucosal healing in IBD is associated with decreased rates of hospitalization, surgery, steroid use and risk of malignancy (4–8).

In the ‘treat to target’ era of IBD management, objective monitoring of disease activity is necessary to guide treatment decisions (4,7–9). International guidelines recommend frequent endoscopic assessment whilst adjusting therapy (4). However, the burden of endoscopy is substantial in terms of patient risks, need for bowel preparation, inconvenience, cost and loss of productivity.

Noninvasive modalities may be useful to objectively monitor IBD disease activity as a surrogate for endoscopy (10). The role of faecal and serum biomarkers is well-established in IBD practice (10–13). Magnetic resonance imaging (MRI) is a useful noninvasive tool for assessing small bowel involvement and distinguishing active from fibrotic strictures when contrast is used (14). Bowel cleansing is required for MRI and it may be limited by cost and accessibility. Gastrointestinal ultrasound (GIUS) is an accurate tool for assessment of IBD disease activity and extent as well as the presence of complications (15). GIUS closely correlates with endoscopic mucosal healing in both ulcerative colitis (UC) and Crohn’s disease (CD) (16), and holds advantages over other tools because it can be performed at the point of care to expedite clinical decision making. The sensitivity of GIUS ranges from 54 to 93%, however it increases to 94 to 100% when oral contrast enhancement is used, and the specificity ranging from 97 to 100% (17). GIUS has been shown to be comparable to MRI and computed tomography (CT) with regards to disease detection, disease extent, and complications such as abscess, fistulas and strictures (18,19). GIUS is accurate and comparable to MRI and video capsule endoscopy in detecting small bowel disease and accurate in diagnosing postoperative recurrence (20,21). CT imaging is generally reserved for acute presentations because of the risk of repeated radiation exposure (22).

Patients with IBD are subject to multiple diagnostic tests over the course of their chronic illness, yet there are few studies exploring patients’ perspective of monitoring tools in IBD. Acknowledgement of patients as ‘consumers’ is increasingly important as the accuracy and comparability of noninvasive imaging tools for IBD assessment have been demonstrated (15,23). The aim of this study was to systematically review the literature on patient perspectives of tools used to monitor disease activity in adult patients with IBD, regarding preference, tolerance and/or acceptability.

## MATERIALS AND METHODS

The review protocol was prospectively registered on PROPERO, CRD42018111311

### Information Sources and Searches

This systematic review followed the PRISMA 2009 guidelines. A systematic search of Embase, Medline, Cochrane Central and Clinical Trials.gov from January 1980 to April 2019 was performed. The detailed search strategies are outlined in Supplementary Appendix 1. All identified papers were catalogued using EndNote X8.

### Study Selection and Eligibility Criteria

Studies were eligible for inclusion in the systematic review if reporting on the perspectives of adult patients with a formal diagnosis of IBD on diagnostic tools for monitoring disease activity, in which two or more monitoring tools or modalities were compared. All study types including randomized controlled trials, cohort studies and observational studies published in abstract or full text were considered. Outcomes of tolerability, patient preference, satisfaction, acceptability, or perception of clinical utility were evaluated. All studies published between January 1980 and April 2019 were eligible. Only studies with English abstracts were reviewed.

Two researchers (T.M.G. and R.N.) independently screened the titles and abstracts and selected articles for full text review. Full text articles were then reviewed for eligibility, with arbitration by a third author (R.B.) for any differences that could not be resolved by consensus. A manual search of the references of eligible studies was also performed.

### Data Extraction and Quality Assessment

Two researchers (T.M.G. and R.N.) independently extracted data from eligible articles. From each article, the first author, journal, year of publication, aim of study, design, funding source, ethical approval, specific outcome measures and numbers in each group, outcomes, and all reported objective results were extracted.

Quality assessment was performed using the Newcastle-Ottawa quality assessment scale for cohort studies (NOS) (Supplementary Appendix 2) (24). The study quality was then graded using the thresholds for converting the NOS to Agency for Health Research and Quality standards as: Good quality (3 or 4 points in selection domain AND 1 or 2 points in comparability domain AND 2 or 3 points in outcome/exposure domain), Fair quality (2 points in selection domain AND 1 or 2 points in comparability domain AND 2 or 3 points in outcome/exposure domain), Poor quality (0 or 1 points in selection domain OR 0 points in comparability domain OR 0 or 1 points in outcome/exposure domain).

### Data Synthesis and Analysis

Outcome measures were determined after study selection due to the heterogeneous nature of the data reported. Relevant data were included from each eligible study, subsequently categorised into domains of patient satisfaction with monitoring tools, patient acceptability of monitoring tools, and patient preference for monitoring tools. Appropriate summary data and descriptive analysis are presented as counts and percentages. Overall patient preference was reported using Pearson’s Chi^2^ test. All analysis was performed using Stata 14.2 (StataCorp, USA).

## RESULTS

### Search Results and Included Studies

**Figure 1.**
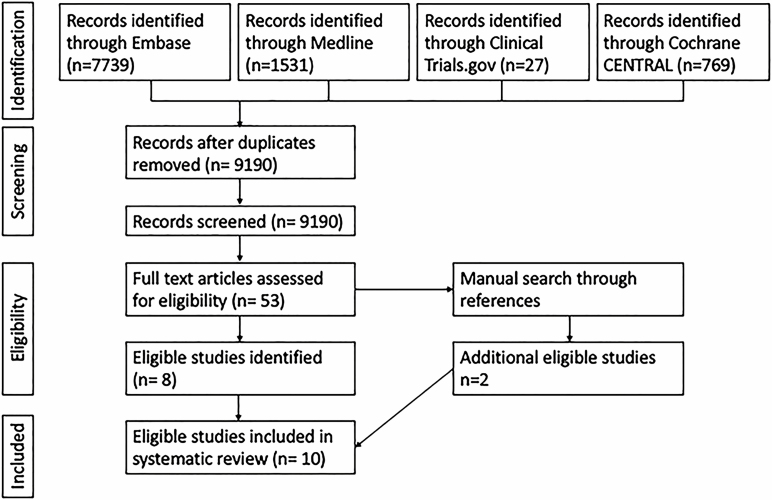
Prisma diagram.

The systematic search yielded 10,073 studies including 883 duplicates, leaving 9190 studies for screening. After the initial screening 53 abstracts were selected for full text review and eight studies were determined to be eligible. A manual review of references yielded a further two eligible studies (25,26) (Figure 1).

### Study Characteristics

Ten studies published between 2005 and 2019, evaluating 1846 participants, were identified (Table 1). All studies were observational in nature. The study size was heterogeneous, ranging from 18 to 916 participants (median 88.5, interquartile range 31–210; mean 185 ± standard deviation 270). One study reported on patients with suspected colorectal cancer, among whom only those with IBD (10/18) were included in the analysis (26). Four studies were available in abstract form only. One Spanish study only had an English abstract and the original study was irretrievable (27). Further information was provided by the authors of two abstracts (28,29). Five studies compared two monitoring tools, two studies compared multiple imaging modalities (28,30) and three studies compared multiple monitoring tools in CD and UC populations (29,31,32).

**Table 1.** Characteristics and results of included studies

Study (year)	Study type	Participant number (disease phenotype)	Participant characteristics	Age—years (mean ± SD)	Monitoring tools compared	Outcome measures	Assessment tool
Buisson et al. (2017) (31)	Cohort	916 (618 CD, 298 UC)	French adults with established IBD. Male 40.4%	CD 38.2 (20) UC 42.1 (14.5)	Venipuncture Stool collection Colonoscopy Rectosigmoidoscopy MRE^†^ Ultrasound^†^ WCE^†^	Acceptability	100 mm VAS
Camara Viudez et al. (2014) (27)	Cohort—abstract only	48 CD	Spanish adults with CD. Male 50%	43 (13.8)	Colonoscopy MRI colonography	Preference	Binary ranking
Chang et al. (2011) (33)	Cohort—abstract only	27 Majority CD	Australian adults with IBD requiring colonoscopy Male 63%	39	Colonoscopy FC	Satisfaction Preference	Likert scale and binary preference ranking.
Florie et al. (2005) (25)	Cohort	31 CD	Dutch adults with suspected CD relapse. Male 71%	36 (12)	Colonoscopy MRE	Preference	Likert scale and binary preference ranking.
Friedman et al. (2018) (28)	Cohort—abstract and unpublished data only	260 (GIUS = 73, non-GIUS = 187)	Australian adults with IBD. Male 53.8%	GIUS 38.8 (13.8) Non-GIUS 40 (12.4)	GIUS Colonoscopy CT MRI	Satisfaction Preference	100 mm VAS and preference ranking
Hafeez et al. (2012) (26)	Cohort	18 (10 IBD [2 CD, 8 UC], 8 non-IBD and excluded from analysis)	English adults enrolled in a trial comparing MRI colonoscopy with colonoscopy. Male 61%	41 (range 17–65)	Colonoscopy MRI colonoscopy	Preference	Binary ranking
Lahat et al. (2016) (34)	Cohort	56 CD	Israeli adults with small bowel CD, mild disease or in remission Male 59%	32 (11)	WCE MRE	Preference	Binary ranking
Miles et al. (2019) (30)	Cohort	159 CD	UK patients aged 16 years and over with a new diagnosis of CD or established CD with suspected flare. Male 40.9%	38.2 (16.4)	Colonoscopy Rectosigmoidoscopy MRE GIUS Hydro-GIUS CT-Enterography BaFT	Acceptability Burden Preference	Likert scale and binary preference ranking for MRE and GIUS
Noiseux et al. (2019) (32)	Cohort	210 (145 CD, 65 UC)	Canadian adults with IBD and members of Crohn’s and Colitis Canada. Male 18.6%	Unclear	General blood test Stool test Colonoscopy Colon biopsy Medical imaging	Level of comfort	Likert scale
Rajagopalan et al. (2018) (29)	Cohort—abstract and unpublished data only	121 (79 CD, 42 UC)	Australian adults with IBD undergoing GIUS during routine clinical care. Male 45%	42 (17)	Blood sampling Stool sampling Colonoscopy Sigmoidoscopy GIUS Imaging^†^	Acceptability	10-point VAS

BaFT, barium follow through; CD, Crohn’s disease; FC, faecal calprotectin; GIUS, gastrointestinal ultrasound; IBD, inflammatory bowel disease; MRE, magnetic resonance enterography; MRI, magnetic resonance imaging; WCE, wireless capsule endoscopy; SD, standard deviation; UC, ulcerative colitis.

†=CD cohort only.

### Study Quality

One study was classified as good quality, four studies as fair quality and five studies as poor quality (Table 2). Common domains for low quality assessment were ascertainment of exposure and assessment of outcome as all, but one study used a self-written survey. In all studies, the participants had a risk of prior exposure to the outcome. Two studies had inadequate follow-up with 48% and 22.5% response rates (30,32).

**Table 2.** Newcastle-Ottawa Scale assessment of study quality

Study (year)	Selection				Comparability	Outcome			Total score (/9)	AHRQ Standard
	Representative-ness of the exposed cohort (/1)	Selection of the non-exposed cohort (/1)	Ascertainment of exposure (/1)	Demonstration that outcome of interest was not present at the start of study (/1)	Comparability of cohorts on the basis of the design or analysis (/2)	Assessment of outcome (/1)	Was follow-up long enough for outcomes to occur (/1)	Adequacy of follow-up (/1)		
Buisson et al. (2017) (31)	1	1	0	0	2	0	1	1	6	Fair
Camara Viudez et al. (2014) (27)	1	1	0	0	1	0	1	0	4	Poor
Chang et al. (2011) (33)	1	1	0	0	2	0	1	1	6	Fair
Florie et al. (2005) (25)	1	1	0	0	2	0	1	1	6	Fair
Friedman et al. (2018) (28)	1	1	0	0	0	0	1	1	4	Poor
Hafeez et al. (2012) (26)	1	1	1	0	2	0	1	1	7	Good
Lahat et al. (2016) (34)	1	1	0	0	2	0	1	1	6	Fair
Miles et al. (2019) (30)	1	1	0	0	2	0	1	0	5	Poor
Noiseux et al. (2019) (32)	1	1	0	0	2	0	1	0	5	Poor
Rajagopalan et al. (2018) (29)	1	1	0	0	2	0	1	1	6	Fair

### Satisfaction with IBD Monitoring Tools

Overall, two studies evaluating 287 patients with IBD both reported higher satisfaction with noninvasive tools. Chang et al. (2011) reported overall satisfaction ratings from 27 patients undergoing FC (faecal calprotectin) testing and colonoscopy on a standardized five-point scoring scale, with a trend towards greater satisfaction for FC (4.11) over colonoscopy (3.51), although the difference was not significant (*P* = 0.069) (33). Friedman et al. (2018) reported satisfaction scores with IBD imaging techniques (GIUS, CT, MRI and colonoscopy) from 260 IBD patients in clinic using a 100 mm visual analogue scale (VAS). GIUS was rated the highest level of satisfaction (90.9) (28) (Table 3).

**Table 3.** Patient satisfaction, acceptability and preference for monitoring tools in IBD

Study	Monitoring tools compared	Measurement tool	Domain reported		
			Patient satisfaction	Acceptability of monitoring tool	Patient preference
Buisson et al. (2017)	Colonoscopy GIUS MRE Rectosigmoidoscopy Stool collection Venipuncture WCE	100 mm VAS	-	CD: GIUS (9.3) and venepuncture (9.3) most acceptable, WCE (8.5), M RE (8.0), and stool collection (7.7) all similar, colonoscopy (6.7) and rectosigmoidoscopy (4.4) least acceptable (*P* < 0.0001^†^). UC: Colonoscopy (7.5), stool collection (8.1) and venipuncture (9.4) similar, rectosigmoidocopy least acceptable (6.7) (*P* < 0.001)	-
Camara Viudez et al. (2014)	Colonoscopy MRI colonography	Binary ranking	-	-	Trend for preference of Colonoscopy (48%) over MRI (33%) (*P* = 0.13)
Chang et al. (2011)	Colonoscopy FC	Likert scale and binary ranking	Trend towards greater satisfaction with FC compared with colonoscopy (*P* = 0.69)	-	FC preferred over colonoscopy by 92% (*P* < 0.001)
Florie et al. (2005)	Ileocolonoscopy MRE	Likert scale and binary ranking		-	MRE preferred over ileocolonoscopy by 94% (*P* < 0.001)
Friedman et al. (2018)	Colonoscopy CT GIUS MRI	100 mm VAS and preference ranking	GIUS rated highest level of satisfaction. Rating for GIUS in experienced patients (90.9) higher than in treatment naïve (83.7) (*P* = 0.033)	-	GIUS preferred over other modalities by 65% and 62% of GIUS experienced and naïve patients^‡^
Hafeez et al. (2012)	Colonoscopy MRI Colonography	Binary ranking	-	-	Trend for preference of MRI colonography (50%) over colonoscopy (30%) (*P* = 0.36)
Lahat et al. (2016)	MRE WCE	Binary ranking	-	-	WCE preferred over MRE by 78% (*P* < 0.0001)
Miles et al. (2019)	BaFT Colonoscopy CTE Hydro-GIUS GIUS MRE Rectosigmoidoscopy	Likert scale and binary ranking for GIUS and MRE	-	GIUS acceptable in 99% compared with MRE 88% (*P* < 0.001) and lower scan burden (*P* < 0.001). Colonoscopy less acceptable than other tools (*P* < 0.001) Willingness to repeat a test highest for GIUS (99%) compared with MRE (91%) (*P* = 0.012) or colonoscopy (75%) (*P* = 0.017). BaFT, CTE, Hydro-GIUS not different to MRE.	GIUS preferred over MRE by 80% (*P* < 0.0001)
Noiseux et al. (2019)	General blood test Stool test Colonoscopy Colon biopsy Medical imaging	Likert Scale		Percentage reporting high level of comfort for stool test 61.4%, Medical imaging 60.8%, colon biopsy 54.1%, colonoscopy 24.5%, general blood test 9.8%. No statistical comparison available.	
Rajagopalan et al. (2018)	Blood sampling Stool sampling Colonoscopy Sigmoidoscopy GIUS Imaging	100mm VAS		Overall acceptability of GIUS (9.21) was significantly greater than acceptability of blood sampling (8.87), imaging (8.67 CD only), stool sampling (8.17), sigmoidoscopy (8.0 UC only) or colonoscopy (7.94) (*P* < 0.01 for all comparisons).	

BaFT, barium follow through; CD, Crohn’s disease; FC, faecal calprotectin; GIUS, gastrointestinal ultrasound; IBD, inflammatory bowel disease; MRE, magnetic resonance enterography; MRI magnetic resonance imaging; UC, Ulcerative colitis; WCE, wireless capsule endoscopy.

†=For each comparison. ‡=No statistical comparison available.

### Acceptability of IBD Monitoring Tools

Overall, four studies evaluating 1406 patients reported on the acceptability of IBD monitoring tools (Table 3).

Buisson et al. (2017) asked 916 participants with IBD (67% CD) to compare multiple monitoring tools and rate their acceptability on a VAS with 0 the lowest and 10 the highest score. In patients with CD, GIUS and venepuncture were ranked as the most acceptable tools to monitor IBD disease activity (median VAS scores of 9.3 and 9.3, respectively) and were significantly more acceptable than all other tools (*P* < 0.0001). Wireless capsule endoscopy (WCE) (VAS 8.5), magnetic resonance enterography (MRE) (VAS 8.0) and stool collection (VAS 7.7) were all significantly more acceptable than colonoscopy (VAS 6.7) (*P* < 0.0001). Rectosigmoidoscopy was the least acceptable tool (VAS 4.4, *P* < 0.0001). In patients with UC, venepuncture (VAS 9.4), stool collection (VAS 8.1) and colonoscopy (VAS 7.5) were not significantly different in acceptability, however rectosigmoidoscopy was least acceptable (VAS 6.7), *P* < 0.001 (31). GIUS, MRE and WCE were not assessed in the UC cohort.

Miles et al. (2019) also compared multiple monitoring tools on a four-point scale (‘not at all acceptable’ to ‘very acceptable’) among 146 patients with CD. GIUS was considered very or fairly acceptable by 144/146 (99%) of patients, while only 128/145 patients (88%) considered MRE very or fairly acceptable (*P* < 0.001). There was no significant difference in ‘very or fairly acceptable’ rates between MRE, barium follow-through (20/24, 83%), CT enterography (29/31, 94%) or hydro-sonography (41/46, 89%). Colonoscopy rates of ‘very or fairly acceptable’ were significantly lower than other tools (60/100, 60%) (*P* < 0.001). Similarly, the proportion of patients willing to repeat a test was greater for GIUS (133/135, 99%) than for MRE (127/140, 91%, *P* = 0.012) or colonoscopy (68/91, 75%, *P* = 0.017). Willingness to repeat barium follow through, CT enterography and hydro-sonography were not significantly different to MRE (30). Miles 2019 also reported a significantly lower scan burden (composite measure of satisfaction, worry and discomfort derived from a seven-point Likert scale) for GIUS (1.66) compared with MRE (2.72) (*P* < 0.001) (30).

Noiseux et al. (2019) surveyed a Canadian online IBD community. Two hundred and ten out of nine hundred and thirty-three (22.5%) of members responded (69% CD). The six category Likert scale ranged from ‘not at all comfortable’ to ‘very comfortable’ to undergo a test. These were then grouped into three levels of high, medium and low comfort. In order of decreasing rates of comfort, the reported diagnostic and monitoring tests were: stool testing (61.4% high), medical imaging (60.8% high), colon biopsy (54.1% high), colonoscopy (24.5% high) and general blood test (9.8% high). No statistical comparison was made between comfort levels for the different diagnostic and monitoring tests.

Rajagopalan et al. (2018) surveyed 121 patients with IBD (65% CD) undergoing point of care GIUS using a 0 to 10 VAS to rank comparative acceptability of monitoring tools in IBD. Overall, acceptability was greatest for GIUS (9.21), followed by blood sampling (8.87), imaging (8.67, CD cohort only), stool sampling (8.17), colonoscopy (7.94) or sigmoidoscopy (8.0, UC cohort only) (*P* < 0.01 for all comparisons).

### Patient Preference for IBD Monitoring Tools

Seven studies evaluating 432 patients with IBD reported on patient preference of IBD monitoring tools (Table 3). Overall, there was a consistent preference for noninvasive imaging techniques over endoscopy with 75/107 (70.1%, *P* < 0.0001) preferring noninvasive tools (25–28,33). GIUS was also preferred over MRI (28,30). Three studies reported preference for MRI or colonoscopy (25–27) with a total preference for MRI (50/77, 65%) over colonoscopy (27/77, 35%) (*P* < 0.0001).

Camara Viudez et al. (2014) reported that 48 CD patients preferred colonoscopy (23/48, 48%) to MRI colonography (16/48 33%); however, this was not statistically significant (*P* = 0.1344) (27). Chang et al. (2011) found that the majority of *n* = 27 patients (92%) would favour FC over colonoscopy if clinical benefits were identical (*P* < 0.001) (33). Florie et al. (2005) evaluated 31 CD patients with regard to preference for MRE or ileocolonoscopy with sedation, finding that 29/31 (94%) patients preferred MRE over ileocolonoscopy (*P* < 0.001). Significant preference for MRE was consistent across domains of preparation, pain, discomfort and embarrassment (25). Friedman et al. (2018) reported that GIUS was the preferred imaging modality amongst 260 patients with IBD (61% CD) when compared with CT, MRI and colonoscopy (*P* = 0.033), with 65% of patients preferring this modality (28). Hafeez et al. (2012) evaluated 10 patients with IBD (20% CD) demonstrating a nonsignificant preference for MRI colonography (5/10, 50%) over colonoscopy with procedural sedation (3/10 30%) (*P* = 0.3613) (26). Lahat et al. (2016) evaluated 56 CD patients and found a preference for capsule endoscopy over MRE in 44/56 (78%), *P* < 0.0001 (34). Miles et al. (2019) evaluated 159 CD patients and found a preference for GIUS over MRE for small bowel imaging in 100/125 patients (80%), *P* < 0.0001 (30).

## Discussion

This is the first systematic review to evaluate patient perceptions of monitoring tools used in IBD. The key finding was that noninvasive techniques such as FC and GIUS were preferred by patients, as compared to other imaging tools or endoscopy. The small number of quality studies exposes an underappreciation of patients as health care ‘consumers’.

These findings are likely a reflection of the burden that invasive tests place on patients. Colonoscopy necessitates time off work for preparation, procedure and recovery, and can be associated with embarrassment and discomfort, as well as a risk of complication (31). Similarly, the acceptability of CTE and MRE is decreased by the need for intravenous contrast injection, as well as polyethylene glycol preparation, which is associated with faecal urgency and fear of intraprocedural incontinence (31,34). CTE is associated with ionising radiation exposure, which may be cumulative in a young cohort exposed to serial imaging over their disease course (22). WCE requires bowel preparation, carries risk of obstruction and need for surgery, and remuneration is variable between health jurisdictions. The rate of capsule retention and obstruction in established IBD is 8.2% and the burden of a patency capsule test must also be considered when selecting this modality (35).

GIUS was associated with a significantly higher level of satisfaction and acceptability when compared to other imaging modalities, endoscopy or laboratory tests by 63% (1158/1846) of participants in this review (28–31). GIUS is unique in that it can be performed at the point of care, meaning that results are immediately available. GIUS has been shown to have comparable accuracy to both ileocolonoscopy and other imaging modalities in assessing disease activity and extent for both UC and CD (17–21,36,37). Patients can communicate with the examining physician during GIUS, which provides an opportunity for education and generation of rapport (28,29). Two included studies found that IBD patients undergoing GIUS demonstrated significantly greater understanding and knowledge of their disease, with an associated increase in adherence to therapy over time (28,29).

Patients’ perceptions of tools for monitoring IBD are often overlooked yet are an important consideration in therapeutic decision making. Where there is similar accuracy between tests, physician should engage patients in monitoring their IBD. Perhaps the greatest barrier to less invasive/more acceptable monitoring tool use is concern regarding reduced clinical utility.

Clinician perceptions of the utility of monitoring tools in IBD are important and influence the choice of investigations ordered for patients. In centres that utilize routine GIUS and FC, clinicians perceive utility of these tests as equal to MRE and only slightly lower than colonoscopy (31). GIUS is also cheaper than MRE or colonoscopy (38). The uptake of GIUS outside of continental Europe has been slow due in part to a perception of limited clinical utility, operator dependence and limited research data (15,38). FC has been shown to be a valuable early predictor of relapse and disease flares and rises in FC may precede mucosal change (39,40).

This systematic review has some limitations. First, the heterogeneous measurement tools assessed, and different outcomes used in individual studies prevented valid pooling of the data. This partly reflects the absence of any single validated and standardised measurement tool. Buisson et al. (2017) (31) developed an externally reviewed tool which could be further validated for this purpose in the future. Second, paucity of data necessitated a broad capture search strategy. As a result, data of limited quality was evaluated in this systematic review, which may limit generalizability of the findings. Third, the findings are limited to patient perception and do not include clinical utility.

In summary, there is a paucity of data evaluating patients’ perceptions of diagnostic tests in IBD. Existing studies indicate that patients prefer noninvasive and less burdensome diagnostic modalities. Further studies are needed to compare the acceptability of monitoring tools in IBD, as well as their impact on disease-related and health-economic outcomes.

## Author Contributions

Study concept and design was conducted by T.MG., V.J. and R.V.B. Data acquisition was performed by T.M.G., R.N., V.J., T.M.N. and R.V.B.. Data analysis and interpretation was performed by T.M.G., R.N. and R.V.B. Manuscript drafting was performed by T.M.G., S.P.C. and R.V.B. Manuscript editing was performed by T.M.G., R.N., V.J., T.M.N., S.P.C. and R.V.B. Study supervision was performed by R.V.B.

## Conflict of Interest

T.M.G. has no conflicts of interest to declare; R.N. has no conflicts of interest to declare; T.M.N. is an employee of Robarts Clinical Trials Inc; S.P.C. has received grants from NHMRC and research support/consulting fees from Ferring, Janssen, Microbiotica, Shire; Vipul Jairath has received consulting fees from AbbVie, Eli Lilly, GlaxoSmithKline, Arena pharmaceuticals, Genetech, Pendopharm, Sandoz, Merck, Takeda, Janssen, Robarts Clinical Trials Inc., Topivert, Celltrion; Speaker fees from Takeda, Janssen, Shire, Ferring, Abbvie, Pfizer; Robert V Bryant is employed by CALHN/TQEH; Grant/research support/speaker fees (all paid to employer for research support) from AbbVie, Ferring, Janssen, Shire, Takeda, Emerge Health.

## Supplementary Material

gwaa001_suppl_Supplementary_Appendix
