# Supplementary material for: Systematic Review: Patient Perceptions of Monitoring Tools in Inflammatory Bowel Disease
Source: J Can Assoc Gastroenterol. 2020 Jan 24;4(2):e31–41. doi: 10.1093/jcag/gwaa001 (PMC8023822; doi:10.1093/jcag/gwaa001)
Supplement: gwaa001_suppl_Supplementary_Appendix [file gwaa001_suppl_supplementary_appendix.docx]

**Appendix 1. Search Strategy**

**EMBASE**

| 1 | Exp Inflammatory bowel disease/ or exp colitis, ulcerative/ or exp crohn disease/ |
| --- | --- |
| 2 | Exp diagnosis/ or exp "diagnostic techniques and procedures"/ |
| 3 | Magnetic Resonance Imaging/ |
| 4 | Tomography, X-Ray Computed/ |
| 5 | Ultrasonography/ |
| 6 | Colonoscopy/ |
| 7 | Leukocyte L1 Antigen Complex/ |
| 8 | Feces/ |
| 9 | C-Reactive Protein/ or CRP.mp. |
| 10 | Phlebotomy/ |
| 11 | Diagnostic tool*.mp. |
| 12 | Diagnostic modal*.mp. |
| 13 | Diagnostic test*.mp. |
| 14 | Monitor*.mp. |
| 15 | Endoscop*.mp. |
| 16 | CT*.mp. |
| 17 | Venepuncture.mp. |
| 18 | (Calprotectin or Fecal calprotectin).mp. |
| 19 | Stool biomarker*.mp. |
| 20 | Or/2-19 |
| 21 | Exp Patient satisfaction/ |
| 22 | Exp Patient preference/ |
| 23 | Exp "Patient Acceptance of Health Care"/ |
| 24 | Exp Patient comfort/ |
| 25 | Tolerab*.mp. |
| 26 | Acceptab*.mp. |
| 27 | Patient objective*.mp. |
| 28 | Utili*.mp. |
| 29 | Or/21-28 |
| 30 | 1 and 20 and 29 |

**MEDLINE**

| 1 | Exp Inflammatory bowel disease/ or exp colitis, ulcerative/ or exp crohn disease/ |
| --- | --- |
| 2 | Exp diagnosis/ or exp diagnostic techniques and procedures/ |
| 3 | Magnetic Resonance Imaging/ |
| 4 | Tomography, X-Ray Computed/ |
| 5 | Ultrasonography/ |
| 6 | Colonoscopy/ |
| 7 | Leukocyte L1 Antigen Complex/ |
| 8 | Feces/ |
| 9 | C-Reactive Protein/ or CRP.mp. |
| 10 | Phlebotomy/ |
| 11 | Diagnostic tool*.mp. |
| 12 | Diagnostic modal*.mp. |
| 13 | Diagnostic test*.mp. |
| 14 | Monitor*.mp. |
| 15 | Endoscop*.mp. |
| 16 | CT*.mp. |
| 17 | Venepuncture.mp. |
| 18 | (Calprotectin or Fecal calprotectin).mp. |
| 19 | Stool biomarker*.mp. |
| 20 | Or/2-19 |
| 21 | Exp Patient satisfaction/ |
| 22 | Exp Patient preference/ |
| 23 | Exp "Patient Acceptance of Health Care"/ |
| 24 | Exp Patient comfort/ |
| 25 | Tolerab*.mp. |
| 26 | Acceptab*.mp. |
| 27 | Patient objective*.mp. |
| 28 | Utili*.mp. |
| 29 | Or/21-28 |
| 30 | 1 and 20 and 29 |

**Pubmed**

Ti/ab((Inflammatory bowel disease OR IBD OR Ulcerative colitis OR UC OR Crohn’s Disease OR CD)) AND

Ti/ab((Diagnosis OR Diagnostic technique OR Diagnostic procedure OR Magnetic Resonance Imaging OR MRI OR Computed tomography OR Ultrasonography OR Colonoscopy OR Endoscopy Diagnostic tool OR Diagnostic modality OR Diagnostic test OR Venepuncture OR Fecal calprotectin OR Calprotectin OR Stool biomarker )) AND

Ti/ab(( Patient satisfaction OR Patient preference OR Patient acceptance OR Patient comfort OR Clinician preference OR Tolerab* OR Acceptab* OR Utili* OR Satisfaction OR preference* OR Comfort* OR Objective*))

**Cochrane CENTRAL**

| #1 | MeSH: [Inflammatory bowel disease] explode all trees |
| --- | --- |
| #2 | Crohn Disease |
| #3 | Crohn |
| #4 | IBD |
| #5 | Ulcerative colitis |
| #6 | UC |
| #7 | #1 or #2 or #3 or #4 or #5 or #6 |
| #8 | MeSH: [Diagnosis] explode all trees |
| #9 | Magnetic resonance imaging |
| #10 | Computed Tomography |
| #11 | Ultrasonography |
| #12 | Colonoscopy |
| #13 | Leukocyte L1 Antigen Complex |
| #14 | Feces |
| #15 | C-Reactive Protein or CRP |
| #16 | Phlebotomy |
| #17 | Diagnostic tool or Diagnostic modal* or Diagnostic test* |
| #18 | Venepuncture |
| #19 | Calprotectin or Fecal calprotectin |
| #20 | Stool biomarker* |
| #21 | #8 or #9 or #10 or #11 or #12 or #13 or #14 or #15 or #16 or #17 or #18 or #19 or #20 |
| #22 | MeSH: [Patient satisfaction] explode all trees |
| #23 | Patient preference |
| #24 | Patient Acceptance of Health Care |
| #25 | Patient comfort |
| #26 | Patient objective* |
| #27 | #22 or #23 or #24 or #25 or #26 |
| #28 | #7 and #21 and #27 |

**Clinical trials.gov**

1. Inflammatory bowel disease and patient preference
2. Inflammatory bowel disease and patient satisfaction
3. Inflammatory bowel disease and patient acceptance
4. Inflammatory bowel disease and Diagnostic testing

**Appendix 2. Newcastle-Ottawa Scale for Cohort Studies and Thresholds for converting the NOS to AHRQ standards.**
